# Therapeutic drug monitoring of LAI antipsychotics as a predictor of clinical relapse: a one-year follow-up

**DOI:** 10.1192/j.eurpsy.2022.520

**Published:** 2022-09-01

**Authors:** G. D’Anna, F. Rotella, A. Ballerini, V. Ricca

**Affiliations:** University of Florence, Department Of Health Sciences, Florence, Italy

**Keywords:** schizophrénia, LAI, Relapse, Therapeutic drug monitoring

## Abstract

**Introduction:**

Clinical relapses in schizophrenia remain a frequent event. Long-acting injectable (LAI) antipsychotics enhance adherence, but low blood levels can sometimes be observed despite an adequate posology. Nonetheless, the evaluation of this parameter is uncommon in clinical practice.

**Objectives:**

To explore the potential advantages of therapeutic drug monitoring (TDM) of LAIs as a predictor of relapse in clinically stable outpatients with schizophrenia.

**Methods:**

44 individuals who had reached the pharmacokinetic steady state of LAI treatment (paliperidone, olanzapine, aripiprazole) underwent an anamnestic and psychopathological assessment. LAI blood levels were measured using liquid chromatography-mass spectrometry and classified as “in range” or “under range” according to the *Arbeitsgemeinschaft für Neuropsychopharmakologie und Pharmakopsychiatrie* (AGNP) guideline values. Individuals who relapsed during the one-year follow-up were compared to non-relapsers (Fisher’s exact test, χ^2^ or Mann-Whitney U). An exploratory binary logistic regression tested the role of other possible relevant predictors of relapse.

**Results:**

No differences were observed in baseline use of mood stabilisers (p=0.211), antidepressants (p=0.530), or prescribed LAI (p=0.563). Other comparisons are presented in the table: among these variables, in-range LAI levels were the only significant predictor of relapse (F=5.95, p=0.015; OR 0.04, 95%CI 0.02-0.56).

**Table:** 

	Relapse (n=6)	No relapse (n=38)	p
Age (years)	41.33±10.78	43.95±12.98	0.667
Male	4 (66.7%)	21 (55.3%)	0.600
Illness duration (years)	21.83±2.64	19.13±11.82	0.289
Previous acute episodes	3.50±1.05	3.29±1.47	0.652
PANSS-total	49.33±14.83	42.74±14.14	0.231
In-range LAI	2 (33.3%)	32 (84.2%)	0.006

**Conclusions:**

TDM of LAIs may optimise the clinical management of schizophrenia by highlighting a suboptimal dosage and a consequent higher relapse risk. Large-scale, drug-specific assessments are needed to confirm these findings.

**Disclosure:**

No significant relationships.

